# Nucleotide spacing distribution analysis for human genome

**DOI:** 10.1007/s00335-021-09865-5

**Published:** 2021-03-15

**Authors:** Andrzej Z. Górski, Monika Piwowar

**Affiliations:** 1 Polish Academy of Sciences, Institute of Nuclear Physics, Radzikowskiego 152 st, 31-342 Kraków, Poland; 2 Jagiellonian University, Collegium Medicum, Kopernika 7E st, 31-034 Kraków, Poland

## Abstract

The distribution of nucleotides spacing in human genome was investigated. An analysis of the frequency of occurrence in the human genome of different sequence lengths flanked by one type of nucleotide was carried out showing that the distribution has no self-similar (fractal) structure. The results nevertheless revealed several characteristic features: (i) the distribution for short-range spacing is quite similar to the purely stochastic sequences; (ii) the distribution for long-range spacing essentially deviates from the random sequence distribution, showing strong long-range correlations; (iii) the differences between (A, T) and (C, G) nucleotides are quite significant; (iv) the spacing distribution displays tiny oscillations.

## Introduction

The Human Genome (HG) Project was launched in 1990 and was declared complete in 2003. The reference sequence for the HG was sequenced across all chromosomes. Understanding the coding and explanation of the reading of the genetic information contained in the full genomic sequence in view of the enormity of the data—despite analytical efforts—is still a great challenge (Green et al. 2015) (Green et al. 2015). Many studies have proven that the distribution of nucleotides, as well as whole sequences in the human genome is not random as it results from the non-random distribution of coding sequences (genes), CpG regions, as well as regulatory, splice and other functional regions (Denisov et al. 2015) (Majewski and Ott 2002) (Majewski and Ott 2002) (Louie et al. 2003) (Piwowar et al. 2006). Fragments that do not encode in human DNA also have their distinctive distribution profile for specific nucleotides (Babarinde and Saitou 2016) (Sotero-Caio et al. 2017). The aim of many investigations has been to pinpoint important structural characteristics of DNA. For example, local irregularities along a DNA strand, compared to surrounding regions, have been associated with biological functionality (Pinkus 1965). On the other hand, it has been established that the regularity of DNA recording is characterized, for example, by fragments of introns. The coding regions in DNA are irregular (Woods et al. 2016). Exon and intron sequences can be identified from trends of the ratio of the 3-nucleotides periodicity to the background noise in the DNA sequences (Zhao et al. 2018). Computation of regularities has been also applied to biological weighted sequences (strings in which a set of letters may occur at each position with respective probabilities of occurrence) to indicate functionally significant fragments of DNA (Iliopoulos 2005). The above facts indicate that the analysis of nucleotide sequences is still a big challenge and any advance in describing DNA might provide a valuable insight. In this paper the (linear) spacing distribution of each of four nucleotides in the Hunan Genome is analyzed.

The motivation for the presented in the paper analysis was to check to what extent the distribution of nucleotides spacing in the human genome is irregular, taking into account our assumptions. We wanted to check where is the point at which the irregularity of the distribution is clearly observed. We start with the investigation of possible self-similar (fractal) patterns and proceed with statistical distribution of the nearest neighbor spacing for all four nucleotides constituting the genome. This type of analysis of data distribution is widely used not only in physics but also in other sciences, ranging from biomedical (Sotero-Caio et al. 2017) to economical (Górski and Skrzat 2006) applications.

## Materials and methods

The Human Genome (HG) sequence has been taken from the HG Project in the FASTA format (https://www.ncbi.nlm.nih.gov/grc/human/data?asm=GRCh38.p10) (Genome Reference Consortium, Human Reference 2017). It includes the whole HG that is about 3 GB large and contains about 2 billions of nucleotides in chromosome’s fragments. The original text file is converted into numerical files with series of positions of particular nucleotides, A, C, G or T, while the other codes were ignored. The files with concatenated chromosomes are investigated to reveal averaged global properties of Human Genome and they are the starting point for further calculations. It should be stressed that the concatenation has negligible effect on the results because the number of chromosomes as well as the largest spacings are of order 10^2^ while the total length of the HG is of order 10^9^.

## Fractal analysis

First, the possible generalized fractal dimensions (Mandelbrot 1982) of linear distributions of nucleotides A, C, G, T have been calculated. Such calculations, especially when done with a software that cannot be fully controlled, can give misleading results [see, e.g. (Górski and Skrzat 2006) (Górski 2001) (Górski et al. 2016)]. Hence, the calculation has been done with care, using our own box-counting algorithm code, based on the standard formula for the generalized fractal dimension (Mandelbrot 1982) (Górski 2001).

1 $$ d_{q} = \frac{1}{1 - q}\mathop {\lim }\limits_{N \to \infty } \frac{{log\mathop \sum \nolimits_{i} p_{i}^{q} \left( N \right) }}{\log N} = \frac{\log Y\left( N \right) }{{\log N}} $$

where *N* is the number of (linear) divisions, parameter *q* in our case was taken: *q* = 0, 1, 2, for capacity, information and correlation dimensions, respectively; *p*_*i*_*(N)* is number of data points found in *i*-th box for a given division *N*. The generalized fractal dimension (*d_q*) is extracted from the plot of log *Y(N)* vs. log *N*, as a slope of the linear fit.

**Fig. 1:**
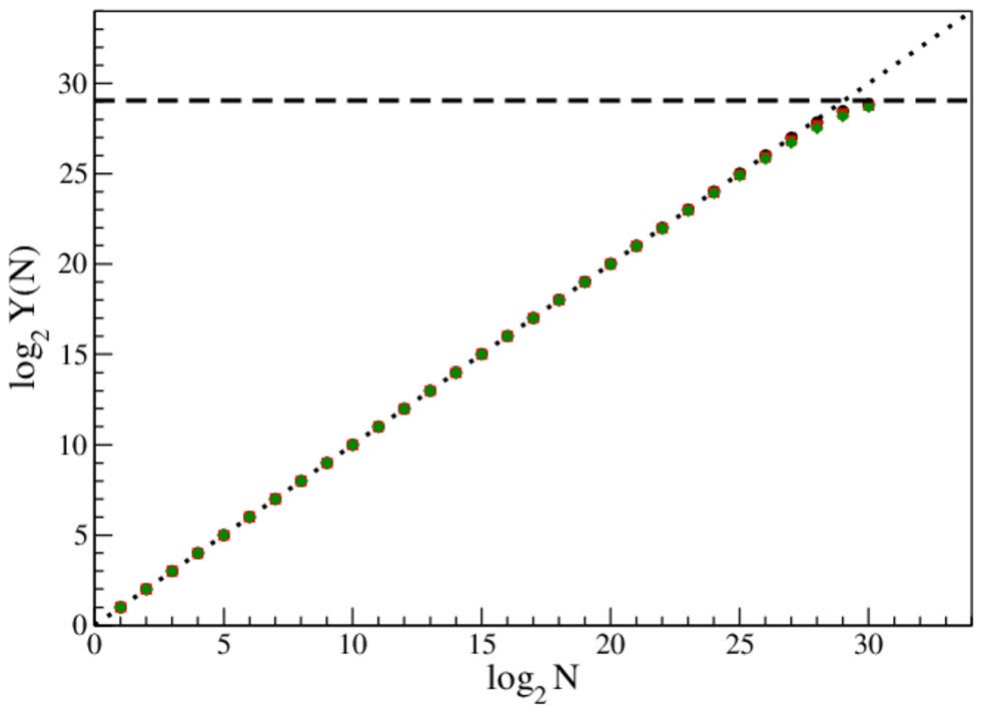
Log–log plot for distribution of base A. Circles, squares and diamonds are for capacity (d0), information (d1) and correlation (d2) dimension, respectively (they strongly overlap). The dotted line has the slope coefficient equal 1, like for homogeneously or randomly distributed data points. The dashed line shows the saturation limit for the ordinate, due to the finite size of the data sample

The resulting standard log–log plot used to extract generalized fractal dimensions for nucleotide A is shown in Fig. 1. Circles, squares and diamonds are for capacity (*d*_*0*_), information (*d*_*1*_) and correlation (*d*_*2*_) dimension, respectively. In fact, the three symbols can hardly be distinguished, as they almost perfectly overlap. The names capacity, information and correlation dimension are traditionally used for the parameter *q* = 0, 1 and 2, respectively (Mandelbrot 1982).

For multifractals *d*_*q*_ for different *q*'s have different values, while for fractals *d(q)* is constant.

This excludes multifractality. Moreover, they are placed along the dotted line that has the slope coefficient equal 1.00, like for homogeneously or randomly distributed data points. The dashed line shows the saturation limit for the ordinate, *log*_*2*_*(n*_*dp*_*)*, where *n*_*dp*_ is the total number of data points due to the finite size of the sample (Górski 2001). Figure 1 gives results for the nucleotide A, only. However, identical plots were obtained for all four nucleotides, as well as for selected single chromosomes. Moreover, almost identical plots were obtained for randomly generated data samples of the same size.

Figure 1 implies that the data set has integer (non-fractal) dimension precisely equal to 1.00. Clearly, due to the Hentschel-Procaccia inequality (Hentschel and Procaccia 1983) *d(q)* = 1.00 for all *q* < 2, as the function *d(q)*) is monotonic. Calculations for higher values of *q* were not performed because for very small *p*_*i*_*(N)* in sum in *Eq. *1 their high powers are beyond any reasonable compiler accuracy. Hence, one has to conclude that the spacing distribution of nucleotides in Human Genome does not show any trace of direct self-similarity, fractal or multifractal structure.

In this place, it is worth to remind, that within the 2-dimensional Chaos Game Representation (*CGR*) of DNA sequences (Jeffrey 1990) their fractal structure is well established by many authors (see, e.g. (Moreno et al. 2011)). Self-similarity in those cases is due to the special properties of the *CGR* transformation, that is a kind of recurrence plot technique (Eckmann et al. 1987). These techniques are useful as randomness tests for random number generators (Jeffrey 1990), as well as stationarity tests for time series (Górski and Skrzat 2006). However, they do not imply self-similarity of the data sample by itself. Hence, it should be stressed, that our calculations presented in Fig. 1 are completely different than calculations presented, e.g. in (Moreno et al. 2011) and similar papers. While the cited papers proven the non-stationarity of the data series we have tested its direct fractal properties (of the linear DNA chain). No self-similar structures were found within the linear chain.

Even though the investigated data samples are not self-similar, and they were shown to have high entropy (Schmitt and Herzel 1997)—like random sequences—they are definitely not purely random. This will be shown in the following section. Moreover, even a highly structured data can resemble random series after compression, as the data compression algorithms increase the Shannon entropy.

## Spacing distribution analysis

**Fig. 2:**
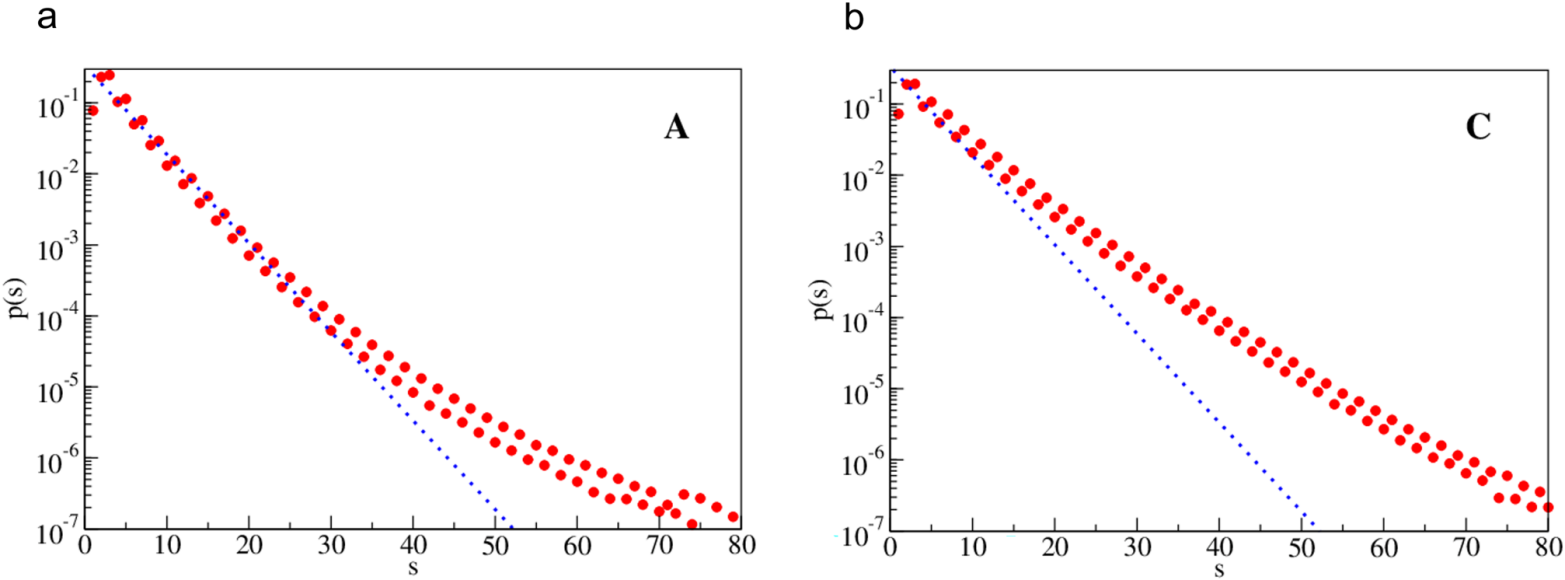
Normalized histogram of spacing distribution, p(s), for bases A and C. The dotted line corresponds to a purely random distribution. Horizontal axis gives spacing distance and the vertical axis gives probability

**Fig. 3:**
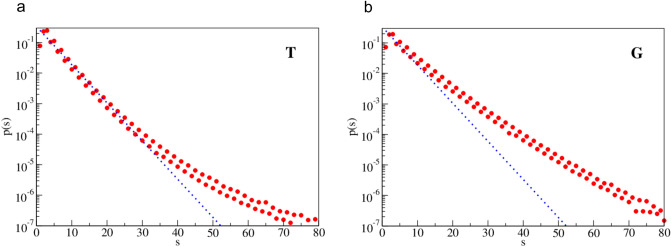
Normalized histogram of spacing distribution, p(s), for bases T and G. The dotted line corresponds to a purely random distribution. Horizontal axis gives spacing distance and the vertical axis gives probability

In this section we analyze the spacing distribution, *p(s)*, between nucleotides of the same type. Here, spacing (*s*) is defined as the distance between two closest neighbors of the same type. For example, for the nucleotide A and the sequence AA the spacing of nucleotides A is *s* = 1. For the sequence AXA, where X is any nucleotide except A, the spacing is *s* = 2, etc. In Figs. 2,3 the circles show (normalized) probabilities, *p(s)*, of a given spacing in the sample. In addition, we added a dotted line that corresponds to the uniform random distribution of nucleotides,

2 $$ p_{ rand} (s) = 1/3x(3/4)^{s} \;,where\sum\nolimits_{s = 1}^{s = \infty } {p(s)} = 1 $$

Such distribution has no long-range correlations and was given as a reference to show the strength of correlations in our case.

For the Human Genome data the spacing distribution has cutoff for s_max_ that is at most of order 103. The total number of occurrences of nucleotide A (and T) is about *5.5* × *10*^*8*^ and for nucleotide C (and G) about *4.1* × *10*^*8*^. In Fig. 2 plots are given for nucleotides A and C, while in Fig. 3 for nucleotides T and G. Both pairs of plots are similar, in accordance with the Chargaff’s rule. All probability distributions are normalized to unity to enable comparison of samples with different sizes.

**Fig. 4:**
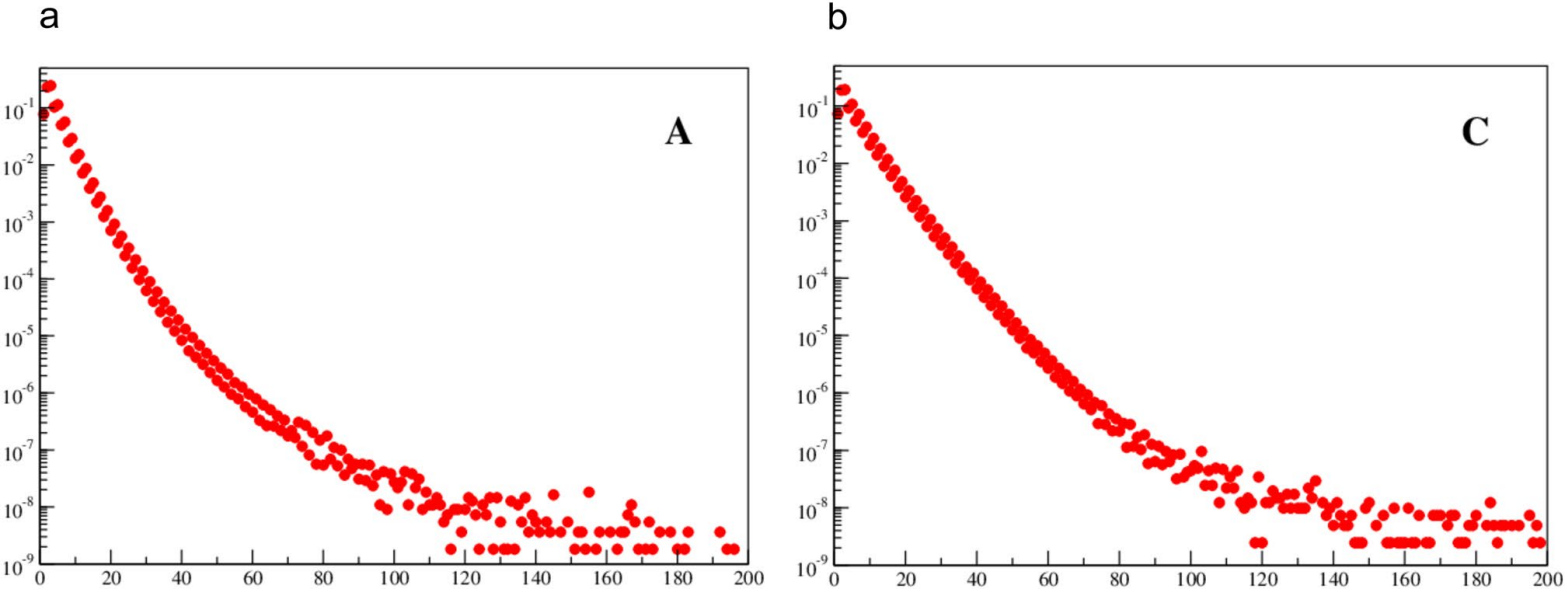
Normalized histogram of spacing distribution for bases A and C with tail up to s = 200. Horizontal axis gives spacing distance and the vertical axis gives probability

**Fig. 5:**
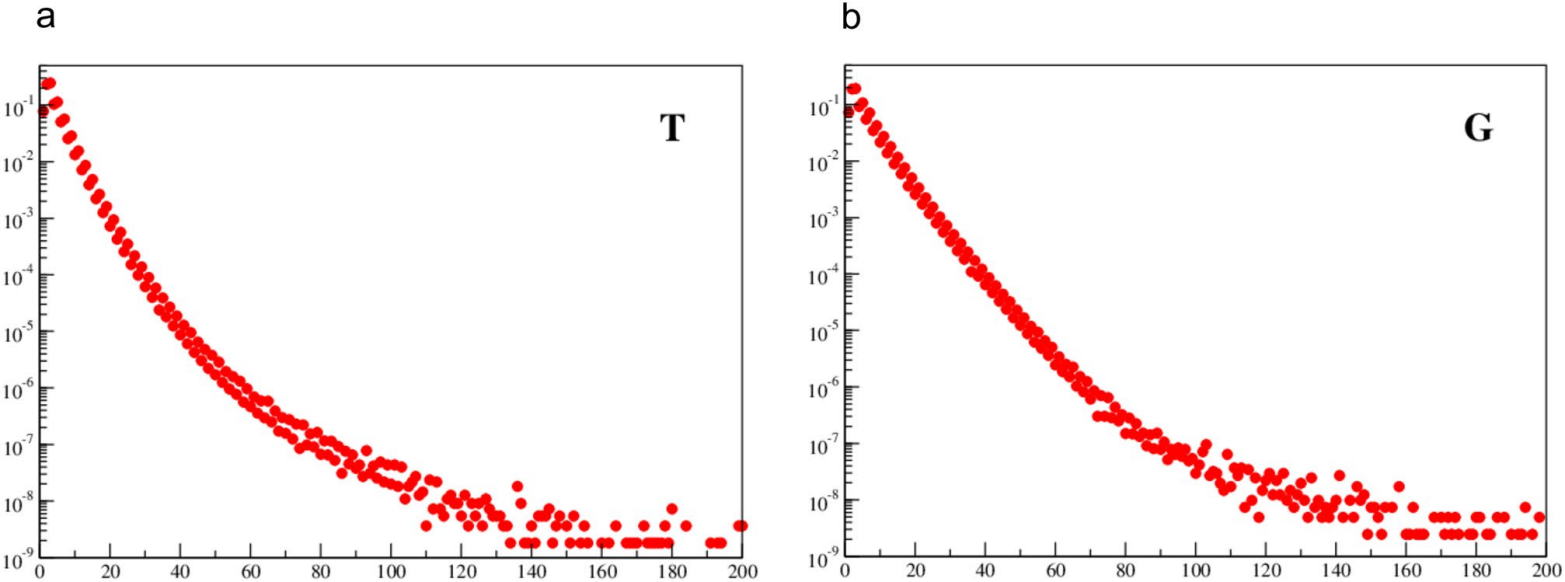
Normalized histogram of spacing distribution for bases T and G with tail up to s = 200. Horizontal axis gives spacing distance and the vertical axis gives probability

In Figs. 4 and 5 the tails of the histograms are shown up to *s* = 200. Here, one can see that for larger spacings (*s*) the tail is getting fat and strongly deviates from exponential behavior. Also, one can see a kind of phase transition at *s*_*2*_ ≈ 80 and the histograms’ bins are more randomly distributed. For *p(s)* approaching 10^−9^ there are only single data points per bin and the statistics becomes less reliable. Hence, though the single events are up to *s* ≈ 1000 they are not displayed. It should be stressed that fat tails are also common for self-organizing systems in economy, sociology, etc., where long-range correlations (*LRC*) occur (Górski and Skrzat 2006).

This phenomenological behavior, though as yet not well understood, seems to be important because of its universality. It was observed for very different systems commonly considered as being complex in economy (Górski et al. 2002), sociology, biology (Górski and Skrzat 2006), linquistic (Lestrade 2017) etc. It is interesting to notice that the characteristic strong correlations and fat tails do occur for distances (*s*) from about 20 up to about 80. It is also unclear why the first threshold is considerably larger for A and T nucleotides than for C and G nucleotides.

Closer examination of spacing distributions reveals several characteristic features that are listed below:

(i) For small spacing (about *s*^*A*^_*1*_ ≈ 30 for A and T nucleotides, but *s*^*C*^_*1*_ < 10 for C and G nucleotides) the distributions are quite close to the purely random distribution. However, for larger spacings the distributions strongly deviate from randomness.
(ii) The long tails of the distributions are strongly enhanced (’fat tails’) in comparison with the random distribution. This suggests strong long distance correlations.
(iii) In general, behavior of nucleotide A is similar as for nucleotide T, and the same holds for the (C,G) pair, though both pairs behave in different way. This can be viewed as another manifestation of the Chargraff’s rule.
(iv) For odd spacing (*s* = 3, 5, 7, …) probability is higher than for their even predecessors. And the difference is slightly higher for (C,G) nucleotides than for (A,T) nucleotides. This is a kind of small high frequency oscillations in the distributions.

## Discussion and conclusion

It has been shown that the nucleotide spacing distribution in the Human Genome is not random, though its high entropy. This is confirmed by the known fact that the nucleotide composition of the DNA sequence determines its spatial structure, function and stability of the spatial structure of the nucleic acid (Vologodskii and Frank-Kamenetskii 2013) (Vologodskii and Frank-Kamenetskii 2018) (Travers 2005).

It has been found that the analyzed distribution has no fractal structure and for small spacings (*s* < *s*_*1*_) it is close to random distribution (exponential decay). Analogous conclusion that the so-called random matches always dominate the distribution for small lengths has also been found recently for eukaryotic genomes (Massip et al. 2015), with similar suggested estimate, *s*_*1*_ ≈ 25. On the other hand, for larger spacing the distribution shows strong correlations and fat tails.

For large distances, *s* > *s*_*2*_ ≈ 80, strong variability around any smooth interpolation was found. Variability of long nucleotide fragments is most likely responsible for structural variation, which is read by molecules interacting with DNA, which are conformationally sensitive. Existence of long-range correlations within the genome of living organism has immense importance in understanding the language of DNA sequences. However, the biological meaning of the long-range correlations in DNA is, as yet, not clear. It is still an open and challenging problem. Long-range correlations suggest that to read the functionality of the human genome, one cannot focus solely on the linear reading of individual nucleotides present in the DNA strand. DNA is a three-dimensional object packed in a specific way in a cell nucleus. DNA is read by unraveling specific DNA fragments in the nucleus space. Probably the interaction of unraveled DNA strand fragments in space may explain the described interactions of long-range DNA fragments. The non-random patterns in DNA with long-range correlation can only be confirmation of this fact.

Research reports that there are non-linear chromatin interactions activating, e.g. transcription factors and long distance DNA interaction (Mifsud et al. 2015)(Noonan and McCallion 2010)(Peng et al. 1992). It confirms the computational observations.

Scientific reports also show a number of other pieces of evidence to explain DNA irregularities and long-range correlations. Long-range correlations (*LRC)* has been suggested to be related to the duplication of DNA fragments. Some authors claim that *LRC* occur only for intron containing DNA sequences, some however, that *LRC* does not distinguish between the intron and intronless DNA sequences. There have also been reports that *LRC* can be related to the nucleosomal structure and dynamics of the chromatin fiber. Our results are in agreement with conclusions reached by other authors, see, e.g. (Massip et al. 2015) (Messer et al. 2007). Moreover, the *LRC* have been shown important to the persistence of resonances of finite segments (Albuquerque et al. 2005).

Attempts are made to analyze the variability of the DNA sequence in terms of structural variation resulting from variation at the sequence level by, e.g. parametric and non-parametric entropy measures. Also, one can speculate, that relatively high entropy of the sequences reported previously (Schmitt and Herzel 1997) (and some similarity to random series) may be an effect of a kind of data compression algorithm. Finally, the A-T and C-G nucleotides have very similar distributions that is in accordance with the Chargaff’s rule. On the other hand, there is clear difference between the two pairs. The C-G nucleotides have significantly higher probability for larger spacing (fatter tails). For *s* = 50 the probability for C is about 10 times higher. On the other hand, the tail for C is shorter and its maximum is slightly higher. Such behavior have also been found for genomes of other species (Afreixo et al. 2009).
